# Severity of radiation pneumonitis, from clinical, dosimetric and biological features: a pilot study

**DOI:** 10.1186/s13014-020-01694-1

**Published:** 2020-10-27

**Authors:** Samantha Aso, Arturo Navarro-Martin, Richard Castillo, Susana Padrones, Edward Castillo, Ana Montes, José Ignacio Martínez, Noelia Cubero, Rosa López, Laura Rodríguez, Ramon Palmero, Federico Manresa, Thomas Guerrero, María Molina

**Affiliations:** 1 Department of Respiratory Medicine, Bellvitge University Hospital; L’Hospitalet de Llobregat, Feixa Llarga S/N, 16th Floor, 08907 Barcelona, Spain; 2 Laboratory of Respiratory Medicine, IDIBELL, Barcelona University; L’Hospitalet de Llobregat, Barcelona, Spain; 3 Department of Radiation Oncology, Catalan Institute of Oncology, L’Hospitalet de Llobregat, Feixa Llarga 199-203, 08908 Barcelona, Spain; 4 Divisions of Radiation Oncology, University of Texas MD Anderson Cancer Center, Houston, TX USA; 5 Department of Computational and Applied Mathematics, Rice University, Houston, TX USA; 6 Department of Nuclear Medicine, Bellvitge Universitary Hospital; L’Hospitalet de Llobregat, Barcelona, Spain; 7 Department of Medical Oncology, Catalan Institute of Oncology: L’Hospitalet de Llobregat, Barcelona, Spain; 8 The University of Texas Health Science Center, Houston, TX USA; 9 CIBER of Respiratory Diseases (CIBERES), ISCIII, Barcelona, Spain

**Keywords:** Non-small cell lung cancer, Radiation pneumonitis, Lung function, Wound healing

## Abstract

**Background and objective:**

Radiation pneumonitis (RP) could be a lethal complication of lung cancer treatment. No reliable predictors of RP severity have been recognized. This prospective pilot study was performed to identify early predictors of high grade lung toxicity and to evaluate clinical, biological or dosimetric features associated with different grades of toxicity.

**Method:**

Sixteen patients with non-small cell lung cancer with indication of concurrent chemoradiotherapy using 60 Gy/2 Gy/fraction starting at cycle one of platinum based chemotherapy were included. Bronchoalveolar lavage (BAL), pulmonary function testing (PFT), and ^18^F-2-fluoro-2-deoxy-D-glucose positron-emission tomography was performed before radiotherapy (RT), after three weeks of treatment, and two months post-RT. For analysis, patients were grouped by grade (low [G1-G2] vs. high [G3-G5]). The two groups were compared to identify predictors of RP. Protein expression BAL and lung tissue metabolism was evaluated in two patients (RP-G1 vs. RP-G3). Categorical variables such as comorbidities, stages and locations were summarized as percentages. Radiation doses, pulmonary function values and time to RP were summarized by medians with ranges or as means with standard deviation. Longitudinal analysis PFT was performed by a T-test.

**Results:**

All 16 patients developed RP, as follows: G1 (5 pts; 31.3%); G2 (5 pts; 31.3%); G3 (5 pts; 31.3%); and G5 (1 pts; 6.1%). Patients with high grade RP presented significant decrease (*p* = 0.02) in diffusing lung capacity for carbon monoxide (DLCO) after three weeks of RT. No correlation between dosimetric values and RP grades was observed. BAL analysis of the selected patients showed that CXCL-1, CD154, IL-1ra, IL-23, MIF, PAI-1 and IFN-γ were overexpressed in the lungs of the RP-G3 patient, even before treatment. The pre-RT SUVmax value in the RP-G3 patient was non-significantly higher than in the patient with RP-G1.

**Conclusions:**

RT induces some degree of RP. Our data suggest that decrease in DLCO% is the most sensitive parameter for the early detection of RP. Moreover, we detect biological differences between the two grades of pneumonitis, highlighting the potential value of some cytokines as a prognostic marker for developing high grade lung toxicity. Further multicenter studies with larger sample size are essential to validate these findings.

## Background

Radiotherapy (RT) is a mainstay treatment for non-small cell lung cancer (NSCLC). Several studies have showed a benefit in local control and survival increasing biological equivalent doses [1]_._ However, its effectiveness is limited by the risk of radiation-induced lung injury (RILI). RILI is the result of an abnormal healing response to lung irradiation caused by damage to parenchymal cells, vasculature, and/or stroma followed by inflammatory cytokine release [2]. Diagnosis of RILI is based on nonspecific symptoms with or without abnormalities in pulmonary function tests (PFT). Radiographic changes usually reveal parenchymal abnormalities.

Radiation pneumonitis (RP), and pulmonary fibrosis (PF) represent, the acute and late phase of RILI. Symptomatic RILI has been described in 30% of cases, with mortality rates as high as 2% [3–6]. Distinctions between these phases is arbitrary because early and late effects of RT are a continuous spectrum of the same biological event. Early-RILI or RP is considered when symptoms appear within 12 week after lung RT and up to 6 months post-RT. X-rays is characterized by inhomogeneous opacity inside or outside the irradiation field and increased density of septal structures. Late-RILI or PF is a chronic lung damage that usually evolves over 6 to 24 months after RT. X-rays shows contracted, dense scar that occupies a much smaller volume than the originally irradiated volume. Also fibroelastosis pleuroparenchymal changes can be observed do to RT [7].

The relationship between the development of RILI and baseline patient characteristics, lung function parameters and radiation dose have been retrospectively investigated [3, 8, 9]. Some molecular biomarkers in blood and bronchoalveolar lavage (BAL) have been proposed [10–12]. In addition, imaging technologies such as ^8^F-2-fluoro-2-deoxy-D-glucose (^18^F-FDG) positron-emission computed tomography (PET/CT) are able to quantify the uptake of ^18^F-FDG in the lung as a marker of pulmonary inflammation [13–17]. Despite these advances, scarce data is available to show reliable predictive factors of RP, the effects of radiation on the lung, or the mechanisms leading to fibrosis and death in the context of RILI.

This prospective pilot study was conducted to identify early predictive factors of severity in RP and to evaluate the possible features associated with different grades of RP.

## Methods and material

### Patients

Patients diagnosed with NSCLC at our Institution with indication of concurrent chemoradiotherapy regimen using 60 Gy 2 Gy/fraction starting at cycle one of platinum based chemotherapy were prospectively included from January 2011 to March 2013. Inclusion criteria comprised: histologically confirmed NSCLC, inoperable locally advanced NSCLC, no previous thoracic RT. Exclusion criteria included: Karnofsky index < 70, interstitial lung disease (ILD), forced expiratory volume at first second (FEV1) < 30%, chronic respiratory failure, oral corticosteroid treatment, contraindication for bronchoscopy, or refusal to participate. The Ethics Committee of the University Hospital of Bellvitge and the Catalan Institute of Oncology approved the study protocol (PR206/08). Patients signed a written informed consent prior to inclusion.

Patients underwent BAL by fiberotpic-bronchoscopy, lung function testing, and ^18^FDG-PET/CT prior to initiation of RT, at the end of the third week of RT, and at two months post-RT. Patient consultations were once weekly from the time of study inclusion until RT completion. Thereafter, patients were evaluated every 15 days for 6 months and then monthly for one year. The follow-up visits included: medical history, physical examination and monthly chest X-rays. RP diagnosis was based on the appearance or worsening of dyspnea and cough, which may associate fever or chest pain, accompanied with changes of radiological images. RP diagnosis and imaging evaluation were made by the multidisciplinary clinical team (medical oncologist, radiation oncologist and thoracic radiologist). RP grade was scored according to the Common Terminology Criteria for Adverse Events version 4.0. (CTCAEv4.0) [18]. Patients were divided into 2 groups (low-grade RP [G1 and G2], and high-grade RP [G3-G5]), according to the CTCAEv4.0. The two groups were compared to identify early predictors for high-grade RP development.

### Radiotherapy treatment

Treatment planning for the RT used a 3D technique. An specific CT scan over the thorax and upper abdomen with intravenous contrast was obtained [19]. Gross tumor volume (GTV) was contoured according to the PET/CT and diagnostic CT scan. No prophylactic nodal irradiation was performed. To cover subclinical disease, the GTV was expanded according to histological findings. The GTV was increased by 0.6 cm for squamous cell carcinomas and by 0.8 cm for adenocarcinomas to provide the clinical target volume (CTV) [20]. The planning target volume (PTV) was determined by adding 0.7 cm to the CTV in the lateral and anterior posterior direction and 1.5 cm in the cranio-caudal direction [21]. The mean dose to the PTV was 60 Gy according to standard protocols [22]. Organs at risk were contoured in accordance with treatment guidelines [23]. Dose constrains to the lungs were V20 < 35% (i.e., 35% of the healthy lung should receive ≤ 20 Gy) with a mean dose < 19 Gy. Radiotherapy and chemotherapy were started at the same time.

### Pulmonary function testing

PFT parameters were measured according to European Respiratory Society guidelines [24] using computerized lung function testing equipment (Body Box 5500; Morgan Scientific). The parameters assessed included forced vital capacity (FVC), forced expiratory volume at first second (FEV1), and diffusing lung capacity for carbon monoxide (DLCO). The same technician performed the PFT at all follow-up consultations.

### BAL sample collection

BAL was performed in both lungs (i.e., the irradiated and non-irradiated) by using four 40 mL aliquots of isotonic saline solution (0.45%) per wash through a fiberoptic-bronchoscope (Olympus BF-160) to facilitate the extraction of cytokines and chemokines from the alveolar space. The first sample was discarded; the second and the third samples were mixed and sent for cytological evaluation. The fourth aliquot was centrifuged (543.6 g × 5 min) into cellular fraction and supernatant, which was aliquoted for cytokine determination; both fractions were frozen at − 80 °C.

### Protein array analysis of cytokine in BAL supernatant

To evaluate differences in individual predisposition to lung damage and tissue repair response, we evaluate BAL samples. In this preliminary report, we chose one representative patient from each study group: one from low grade RP (RP-G1) and another from high grade RP (RP-G3). Expression protein was assessed using the Human Cytokine Array Panel A (R&D Systems, Minneapolis, MN; USA). Protein concentration was measured in each sample [25]. Pixel density of the spots was analysed using the Multi Gauge V3.0 (FujiFilm, Palo Alto, CA; USA). Three independent readers compared the mean pixel density values in the spots.

### Positron-emission computed tomography

Patients underwent PET/CT imaging with ^18^F-FDG according to standard practice [26]. They were asked to fast 6 h prior to the imaging session to ensure fasting blood glucose levels within the normal range (3.3–5.6 mmol/L). Patients received an intravenous administration of FDG per kg of body weight. The ^18^F-FDG-PET/CT scan was performed with a hybrid PET/CT scanner (General Electric Discovery ST). The whole-body acquisition protocol included a CT scan and a PET scan in a three-dimensional mode. No iodine intravenous contrast was administered. The CT data were used for attenuation correction and anatomic location of PET findings. The standardized uptake value (SUV) was used to measure uptake in the lungs.

### Image analysis

Pre-treatment PET/CT image analyses of the two patients (i.e., RP-G1 and RP-G3) were performed to screen for possible differences in the SUV. This analysis was processed by three independent readers and evaluated using custom Matlab software (v2011a, Mathworks, Inc; Natick, MA; USA). The lung region of interest (ROI) was segmented semi-automatically. Overlap of central airway, liver, heart, diaphragm, and tumor in the lung ROI were manually removed. The resulting binary lung ROI was used for the analysis. The SUV was calculated from the PET attenuation corrected emission images [27]. SUV of voxels in the lung ROI were binned into histograms; the maximum SUV (SUVmax) was calculated according to the formula described by Petit el al. [15].

### Statistical analysis

Since the prevalence of RP is variable due to differences in diagnostic scales [4], sample size was calculated using the “observed versus a reference mean” [28], which includes the reported prevalence of RP among the interstitial lung disease [29]. To detect a relevant clinical difference (alpha = 0.05 and beta = 0.1), 17 patients were required (assuming a follow-up loss rate of 20%).

Patients were divided into 2 groups (low-grade [G1 and G2], and high-grade [G3-G5]), according to the CTCAEv4.0. The two groups were compared to identify predictors for the early identification of RILI. Categorical variables were summarized as percentages. Ordinal categorical variables were summarized by medians with ranges or as means with standard deviation (SD). Longitudinal analysis of FEV1(%) and DLCO(%) was performed by a *T*-test. Differences were considered statistically significant for *p* < 0.05. All plots and analyses were performed using the statistical software R version 3.2.1 for Windows.

## Results

### Population

Seventeen patients were invited to participate and one refused. A total of 16 patients were included. Table 1 shows the characteristics of the cohort. All patients developed RP, with different grades of severity distributed as follows: 5 patients, G1 (31.3%); 5 patients, G2 (31.3%); 5 patients, G3 (31.3%); and 1 patient, G5 (6.1%) who died 3 weeks after beginning the treatment. The rest of them had one year of follow-up from the start of concurrent chemoradiotherapy. Patients were grouped by RP grade (low [G1-G2] vs. high [G3-G5]), with 10 and 6 patients in each group, respectively. Four patients from the high-grade group developed RILI in both lungs.

**Table 1:** Clinical and treatment characteristics of the sample

Characteristics	
No. of patients	16
*Sex*	
Male	14 (87.5%)
Female	2 (12.5%)
Age (range)	63 (58.8–76)
*Smoking history*	
Current	10 (62.5%)
Former	5 (31.2%)
Never	1 (6.2%)
*Pulmonary function (range)*	
FVC (%)	101 (87–105.8)
FEV1 (%)	85.5 (71.5–93.3)
FEV1/FVC	67.9 (60.5–73)
DLCO (%)	71 (57.2–87.5)
*Comorbidities*	
Hypertension	5 (31.2%)
Diabetes	3 (18.8%)
COPD	11 (68.8%)
Heart disease	2 (12.5%)
Vascular disease	2 (12.5%)
*Histologic type*	
Adenocarcinoma	4 (25%)
Squamous cell carcinoma	9 (56.2%)
Large cell neuroendocrine carcinoma	2 (12.5%)
NSCLC	1 (6.2%)
*Location*	
Mediastinum	1 (6.2%)
Hilar	3 (18.8%)
Right upper lobe	6 (37.5%)
Right inferior lobe	2 (12.5%)
Left superior lobe	3 (18.8%)
Left inferior lobe	1 (6.2%)
*Radiation doses (range)*	
Mean dose (Gy)	17.2 (12.7–22.9)
V20 (%)	30 (20.3–35.8)
V5 (%)	60 (47.5–65.8)
*Pneumonitis grades*	
1	5 (31.3%)
2	5 (31.3%)
3	5 (31.3%)
4	0 (0%)
5	1 (6.1%)

FVC: forced vital capacity, FEV1_:_ forced expiratory volume in one second, DLCO: diffusing lung capacity for carbon monoxide, COPD: chronic obstructive pulmonary disease, NSCLC: non-small cell lung cancer

Table 2 shows the patient characteristics by RP group (low vs. high grade). There were no significance differences between the groups in terms of age, gender, comorbidities, PFT baseline values, cancer histology, stage and tumor localization. No differences were observed in the time of onset of RP and severity (*p* = 0,6642). The mean radiation dose was higher in the high-grade group [18 Gy (15.2 Gy-20.1 Gy) vs. 16.1 Gy (12 Gy-22.2 Gy)]; however, V20 was lower in the high-grade group [29.5 Gy (95% CI 23–30) vs. 32 Gy (95% CI 21–35)]. No significant correlation between dosimetric values and RP grades was observed.

**Table 2:** Patient characteristics according to pneumonitis grade: low vs. high grade

Characteristics	Low grade (G1–G2)	High grade (G3–G5)
No. of patients	10 (62.5%)	6 (37.5%)
*Sex*		
Male	9 (90%)	5 (83.3%)
Female	1 (10%)	1 (16.7%)
Age (range)	66.0 (59.2–67)	59.5 (58.2–65.2)
*Smoking history*		
Current	8 (80%)	2 (33.3%)
Former	2 (20%)	3 (50%)
Never	0 (0%)	1 (16.7%)
*Pulmonary function (range)*		
FVC (%)	100,5 (88.5- 106.5)	102.5(82.5–104.8)
FEV1 (%)	81.5 (59–93)	89 (77–90.5)
FEV1/FVC	64 (55–71.6)	69.5 (67.9–80.3)
DLCO (%)	71 (46.8–83.5)	71.5 (63.5–92.2)
*Comorbidities*		
Hypertension	3 (30%)	2 (33.3%)
Diabetes	0 (0%)	3 (50%)
COPD	7 (70%)	4 (66.7%)
Heart disease	1 (10%)	1 (16.7%)
Vascular disease	2 (20%)	0 (0%)
*Histologic type*		
Adenocarcinoma	3 (30%)	1 (16.7%)
Squamous cell carcinoma	5 (50%)	4 (66.7%)
Large cell neuroendocrine carcinoma	2 (20%)	0 (0%)
NSCLC	0 (0%)	1 (16.7%)
*Clinical stage*		
IIB	3 (30%)	-
IIIA	6 (60%)	3 (50%)
IIIB	1 (10%)	5 (50%)
*Location*		
Mediastinum	1 (10%)	0 (0%)
Hilar	2 (20%)	1 (16.7%)
Right upper lobe	4 (40%)	2 (33.3%)
Right inferior lobe	1 (10%)	1 (16.7%)
Left superior lobe	1 (10%)	2 (33.3%)
Left inferior lobe	1 (10%)	0 (0%)
*Chemotherapy agents*		
Carboplatin-etoposide	1 (10%)	1 (16.7%)
Carboplatin-gemcitabina	1 (10%)	0 (0%)
Cisplatin-vinorelbine	2 (20%)	0 (0%)
Cisplatina-etoposide	6 (60%)	5 (83.3%)
*Radiation doses (range)*		
Mean dose(Gy)	16.1 (12–22.2)	18 (15.2–20.1)
V20 (%)	32 (21–35.8)	29.5(23–30)
Onset of radiation pneumonitis (median)	68,5 days	111 days

FVC: forced vital capacity, FEV1_:_ forced expiratory volume in one second, DLCO: diffusing lung capacity for carbon monoxide, COPD: chronic obstructive pulmonary disease, NSCLC: non- small cell lung cancer

### Pulmonary function testing

**Fig. 1:**
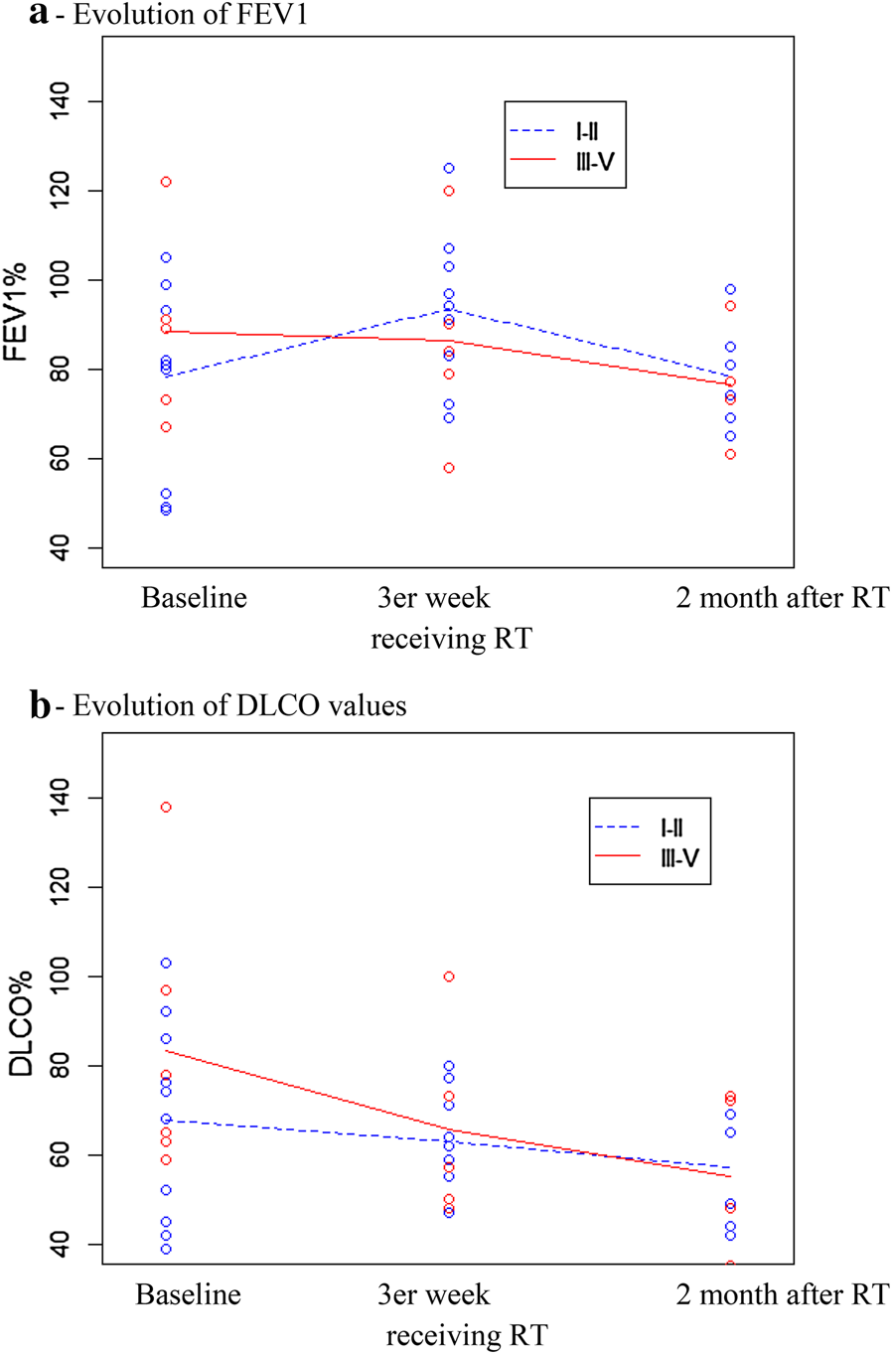
Pulmonary function test values at different time-points by CTCAEv4.0 groups (low-grade [GI and GII] and high-grade [GIII-GV]). **a **Evolution of FEV1: The points represent the FEV1 (%) value of each patient at the three different observation times. The straight, is the mean FEV1 (%) value at each observation time. CTCAEv4.0: Common Terminology Criteria for Adverse Events version 4.0 FEV1: forced expiratory volume in one second RT: radiotherapy. **b** Evolution of DLCO values: The points represent the DLCO (%) value of each patient at three different observation times. The straight line is the mean DLCO (%) value at each observation time. CTCAEv4.0: Common Terminology Criteria for Adverse Events version 4.0 DLCO: diffusing lung capacity for carbon monoxide RT: radiotherapy

Baseline FEV1(%) and DLCO(%) at diagnosis was not associated with the different grade of RP development. No significant differences in mean values were found between the groups at the three time points (baseline, end of week three, and at two month post-RT) (Tables 3 and 4). However, by the end of the third week of RT, the DLCO(%) had decreased substantially in those cases that developed high-grade group (*p* = 0.0203) (Fig. 1-B). Furthermore, the DLCO(%) decline was even worse after 2 months post-RT in that same group (*p* = 0.0342) (Fig. 1-B). A FEV1(%) decline was observed after 2 months of RT but not earlier during the treatment (Fig. 1-A). Therefore, the DLCO(%) decrease during the RT allows to predict a high-grade RP even before starting respiratory symptoms.

**Table 3:** Mean values in FEV1 between CTCAEv4.0 groups at baseline, end of third week, and two month post-RT

Mean values	Low grade (G1–G2)	High grade (G3–G5)	*p *Value
Baseline	78.2% (SD 21.2)	88.5% (SD: 19.1)	0.2803
3 weeks of RT	93.4% (SD 17.5)	86.2% (SD: 22.4)	0.0668
2 months post-RT	78.7% (SD: 12.0)	76.4% (SD 11.8)	0.3015

FEV1: forced expiratory volume in one second, CTCAEv4.0: common terminology criteria for Adverse Events version 4.0, RT: radiotherapy, SD: standard deviation

**Table 4:** Mean values in DLCO between CTCAEv4.0 groups at baseline, end of third week, and two month post-RT

Mean values	Low grade (G1–G2)	High grade (G3–G5)	*p *Value
Baseline	67.7% (SD 22.4)	83.3% (SD: 30.2)	0.0719
3 weeks of RT	62.8% (SD 11.5)	65.6% (SD: 21.6)	0.2136
2 months post- RT	57% (SD: 13.6)	55.2% (SD 16.7)	0.0595

DLCO: diffusing lung capacity for carbon monoxide, CTCAEv4.0: common terminology criteria for adverse events version 4.0, RT: radiotherapy, SD: standard deviation

### Protein array analysis in the BAL supernatant

**Fig. 2:**
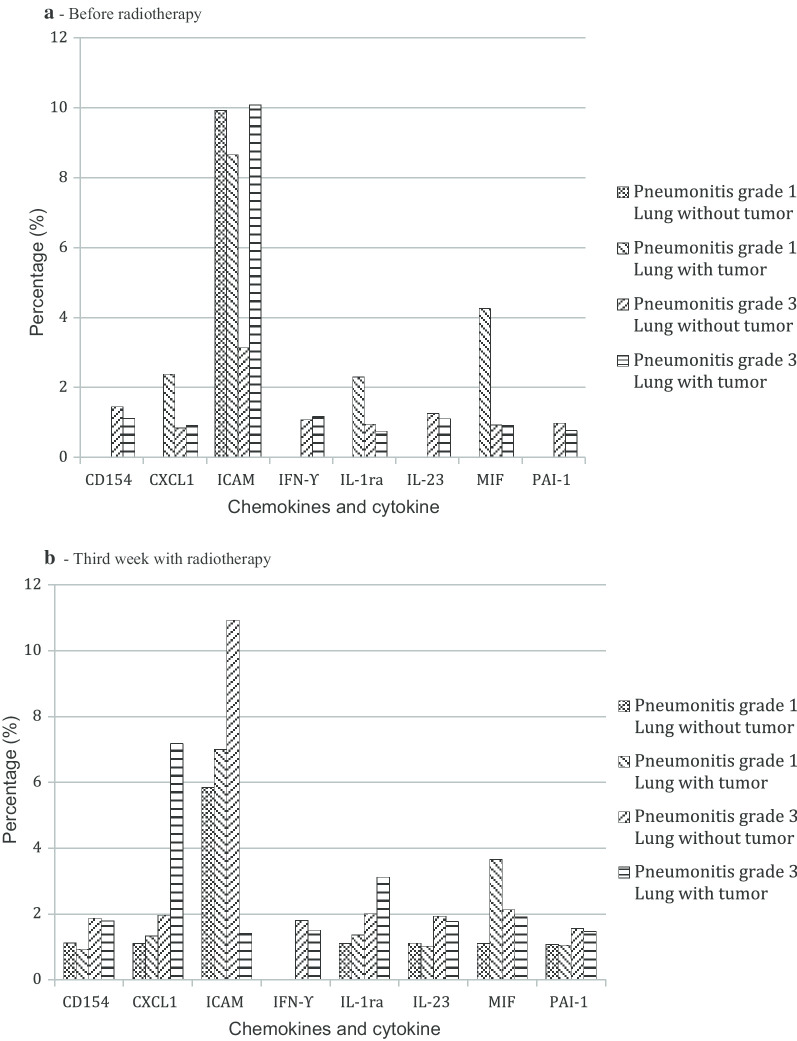
Cytokine and chemokines in bronchoalveolar lavage in patients with grade 1 and grade 3 radiation pneumonitis. **a** Before radiotherapy. **b **Third week with radiotherapy

Different cytokine and chemokine expression profile (pre-RT and week 3) was found in a patient with RP-G1 compared to another with RP-G3 (Fig. 2). Before RT (Fig. 2-A), the only protein expressed in the tumor-free lung of the RP-G1 patient was ICAM while the tumor-free lung of the RP-G3 patient presented an overexpression of CD154, CXCL-1, ICAM, IFN-γ, IL-1ra, IL-23, MIF and PAI-1. In the lung with tumor (Fig. 2-A), the RP-G1 patient expressed CXCL-1, ICAM, IL-1ra and MIF while the RP-G3 patient showed those same cytokines but also expressed CD154, IL-23, IFN-γ and PAI-1. At the end of third week of RT (Fig. 2-B), a change in the cytokine and chemokine patterns was detected in both cases. RT induced expression of CD154, CXCL-1, IL-1ra, IL-23, MIF, and PAI-1 while reducing ICAM expression in both lungs of the RP-G1 patient. RT increased cytokine response of the most overexpressed proteins in the tumor and tumor-free lungs of the RP-G3 patient, with a higher expression of CD154, CXCL1, IL-1ra, IFN-γ, IL-23 and PAI-1 (Fig. 2-B).

### PET/CT image analysis

The pre-RT SUVmax value was calculated for the RP-G1 and RP-G3 patients. In both patients, the pre-treatment SUVmax was higher than normal (standardized lung SUVmax values, 0.05 ± 0.17) [30]. However, the RP-G3 patient had a non-significantly higher SUVmax (2.20 vs. 2, respectively).

## Discussion

The present study demonstrates that RT induces RILI in all patients who undergo external RT with variable clinical manifestations and different degrees of lung damage. This variability in the degree of RILI suggests that severe RP may be associated with differences in individual predisposition. The early decrease of DLCO(%) was a predicting factor of severe RP development and thus could serve as a marker for early diagnosis and treatment modification.

The reported prevalence of RP in lung cancer patients ranges from 0 to 58% [4]. This variation is likely due to differences in diagnostic scales, non-specific symptoms, and the lack of standardized assessment protocols [4]. In the present study, standard follow-up protocols detected RILI in all of the patients but with different severity presentation. This finding suggests that all patients treated with RT are likely to develop RILI to a greater or lesser extent, which may depend on individual biological characteristics.

Previous studies have shown that subtle, local changes in PFT after RT can be used as indicators of acute and chronic lung damage, although published results are not always consistent [3, 9]. The largest and most consistent changes in PFT values after RT are observed in DLCO, which has been directly associated with respiratory morbidity [31]. In the present study, we evaluated the mean differences in FEV1(%) and DLCO(%) between low-grade RP patients [G1-G2] and high-grade RP patients [G3-G5]. We found no statistically significant differences between the two groups in FEV1(%) values at the different time points, thus leading us to conclude that FEV1(%) is not a predictor of RP, a finding that is consistent with other reports [9, 31]. By contrast, we found that a decrease in DLCO(%) 2 month post-RT was predictive of RP severity, in line with some previous reports [9, 31]. Importantly, the decline in DLCO(%) after three weeks of RT was an early predictor of severe RP. The explanatory power of this variable could be that histopathological alterations present during the latency phase (i.e., without clinical manifestations) for RILI may alter gas exchange [3]. Clearly, the ability to early predict severe RILI would be helpful to optimize therapeutic options.

Classically, RP grade has been associated with the radiation dose [3, 8]. In our study, the high-grade group received a higher mean radiation dose but lower V20 than the low-grade group. This finding is contrast with the meta-analysis published by Palma et al. [32] The absence of a significant association in this study between RP grade and radiation parameters could be due to the limited sample size, the small differences among patients with regards to the radiation dose and biological predisposition [9].

The ^18^FDG-PET/CT has been use to quantify increase cell glycolysis in the healthy lung tissue, excluding tumoral areas, like a marker of pulmonary inflammation through the uptake of ^18^FDG mesure by SUV [15-17]. It should be noted that both patients (RP-G1 and RP-G3) presented higher pre-RT SUVmax than normal values (0.05 ± 0.17) [30]. This finding could be explained by the role of chronic inflammation in lung cancer. Tumorgenesis includes a diverse leukocyte cells that has been considered key factors in tumor promotion since they release different variety of cytokines, chemokines, and cytotoxic. These mediators alter the adequate balance between pro-inflammatory and anti-inflammatory cytokines, favoring the increase of the first ones and producing a chronic inflammation state [33].

Accordingly, Castillo et al. [16, 17] demonstrated the predictive value of pre-treatment ^18^F-FDG lung uptake in the subsequent development of RP symptoms. In this study, RP-G3 patient had a higher SUVmax in healthy lung tissue respect RP-G1 patient.These findings support the experience already published by Catillo et al.

RILI produce an imbalance between type 1 and type 2 helper T-cells and abnormal fibroproliferative wound healing [34]. Variations among patients in terms of RP severity and lung repair capacity could be related to individual pre-treatment lung biomolecular conditions and genetic factors [35]. In the present study, increased expression of some mediators were obtained in BAL of tumor lungs in both patients (RP-G1 and RP-G3), although these proteins were also expressed in the tumor-free lung of the RP-G3 patient before RT. Previous studies indicate that IL-1ra is involved in acute inflammation [36]; MIF modulates RILI [37]; and CXCL1 promotes angiogenesis and thus contribute to the pathogenesis of PF. [34] Interestingly, CD154, IFN-γ, IL-23 and PAI-1 were expressed in both lungs (tumor and tumor-free lung) before RT only in the RP-G3 patient. These four cytokines have been described in animal models of lung fibrosis [38, 39]. Furthermore, a recent study reported that a truncated PAI-1 protein protects against RILI in a murine model [40]. Finally, Liu et al. found that rs7242 GT/GG genotypes located in the 3ÚTR of PAI-1 were associated with a significantly increased risk of RP [41]. Overall, our findings suggest a potential biological predisposition to lung damage and altered wound healing in RILI development, which would deserve a depth study to better understand pathogenesis.

### Study strengths and limitations

We have to recognize some limitations: First, the small sample size which was calculated using “observed versus a reference mean” and even although we have enrolled sixteen patients, instead of including seventeen, we have not had any loss of follow up. Secondly, the low power of the biological lung and the PET/CT image analysis (only two patients). In this sense, this is a pilot study to identify if there are differences in biological features associated with different grades of RP. Our findings, warrant further investigation in a larger sample. The main strength of the study is that it is the first prospective study to evaluate patients with NSCLC through a longitudinal clinical and biological follow-up that demonstrate RT induces RILI in all cases but in some of them with a high-grade of lung injury and consequent altered wound repair.

## Conclusion

RT treatment always induces some degree of lung injury and the extent of the damage is variable. Our data suggest that decrease in DLCO% is the most sensitive parameter for the early detection of severe RP. Moreover, we detect biological differences between the two grades of pneumonitis, highlighting the potential value of cytokines such as CXCL-1, CD154, IL-1ra, IL-23, MIF, PAI-1 and IFN-γ as a prognostic marker for developing high grade of lung toxicity. Further multicenter studies with larger sample size are essential to validate these preliminary findings.

## Data Availability

The datasets are available to all interested researchers on reasonable request from corresponding author.

## Abbreviations

CTCAEv4.0: Common terminology criteria for adverse events version 4.0
CXCL1: Chemokine (C-X-C motif) ligand 1
CTV: Clinical target volume
G1: Grade 1
G2: Grade 2
G3: Grade 3
G5: Grade 5
GTV: Gross tumour volume
ICAM: Intercellular adhesion molecular
IFN-γ: Interferon-gamma
IL-23: Interleukin-23
IL-1ra: Interleukin-1 receptor antagonist
MIF: Macrophage migration inhibitory factor
PTV: Planning target volume
PAI-1: Plasminogen activator inhibitor type 1
RT: Radiotherapy
RP-G1: Radiation pneumonitis grade 1
RP-G3: Radiation pneumonitis grade 3
ROI: Region of interest
TGF-β1: Transforming growth factor β-1
V20: Healthy lung that should receive ≤ 20 Gy
